# Supplementary material for: The Systems Biology Research Tool: evolvable open-source software
Source: BMC Syst Biol. 2008 Jun 29;2:55. doi: 10.1186/1752-0509-2-55 (PMC2446383; doi:10.1186/1752-0509-2-55)
Supplement: Additional file 1 — SBRT Archive. An archive of the current version of the Systems Biology Research Tool. [file 1752-0509-2-55-S1.zip › sbrt-1.4.0/doc/developers_guide/api/sbrt/kernel/math/geom/proc/CdHarProcess.html]

CdHarProcess


|  |  |  |
| --- | --- | --- |
| **Overview** \| **Package** \| **Class** \| **Use** \| **Tree** \| **Deprecated** \| **Index** \| **Help** | |  |
| PREV CLASS   NEXT CLASS | **FRAMES**    **NO FRAMES**     **All Classes** |  |
| SUMMARY: NESTED \| FIELD \| CONSTR \| METHOD | DETAIL: FIELD \| CONSTR \| METHOD |  |


---


## sbrt.kernel.math.geom.proc Class CdHarProcess<V>

```
java.lang.Object
  sbrt.kernel.math.geom.proc.CdHarProcess<V>
```

**Type Parameters:**: `V` - the variable type.

**All Implemented Interfaces:**: KernelProcess<CdHarComputer.Results>, MonitoredProcess<CdHarComputer.Results>

---

``` public class CdHarProcess<V> extends java.lang.Object implements MonitoredProcess<CdHarComputer.Results> ```

This class is used to generate random, uniformly distributed points from the
interior of a convex polytope.

**Author:**
:   This class was written and documented by Jeremiah Wright while in the
    Wagner lab.

---

**Constructor Summary**

`CdHarProcess(ConvexPolytope<V> polytope)`
  
          Constructs a new random point generator process for the provided polytope.


| **Method Summary** | |
| --- | --- |
| `ProgressMonitor` | `getProgress()`             Returns a progress monitor for this process. |
| `void` | `run(ResultsManager<CdHarComputer.Results> resultsManager)`             Adds random points to the provided results manager. |
| `void` | `setConstraintTolerance(double tolerance)`             Sets the constraint tolerance. |
| `void` | `setIterations(int iterations)`             Sets the total number of random points to be added to the provided results manager. |
| `void` | `setMaxObstructedMoves(int maxObstructedMoves)`             Sets the maximum number of obstructed moves to allow. |
| `void` | `setMinChordLength(double minChordLength)`             Sets the minimum chord length. |
| `void` | `setPoint(java.util.Map<? extends V,java.lang.Double> startPoint)`             Sets the initial point. |
| `void` | `setPointsPerPoint(int pointsPerPoint)`             Sets the total number of points to generate for each point added to the provided results manager. |
| `void` | `setRandom(java.util.Random random)`             Sets the random number generator. |

**Methods inherited from class java.lang.Object**

`clone, equals, finalize, getClass, hashCode, notify, notifyAll, toString, wait, wait, wait`

**Constructor Detail**

### CdHarProcess

```
public CdHarProcess(ConvexPolytope<V> polytope)
```

:   Constructs a new random point generator process for the provided polytope.

    **Parameters:**: `polytope` - the polytope from which random points will be generated.


**Method Detail**

### setPoint

```
public void setPoint(java.util.Map<? extends V,java.lang.Double> startPoint)
```

:   Sets the initial point. This method sets the initial interior point from
    which the algorithm is started.

    :   **Parameters:**: `startPoint` - variables mapped to their numerical values. **Throws:**: `InvalidPointException` - if the provided point does not lie within the polytope.

---


### setRandom

```
public void setRandom(java.util.Random random)
```

:   Sets the random number generator.

    :   **Parameters:**: `random` - the random number generator with which all psuedorandom numbers are to be produced.

---


### setConstraintTolerance

```
public void setConstraintTolerance(double tolerance)
```

:   Sets the constraint tolerance. This is the amount by which constraints
    can be violated.

    :   **Parameters:**: `tolerance` - the amount by which constaints can be violated. This should be a small positive number, like 1E-9 for example.

---


### setMinChordLength

```
public void setMinChordLength(double minChordLength)
```

:   Sets the minimum chord length. If a computed chord is shorter than the
    provided amount, the move is considered obstructed. This can happen if the
    random walker gets stuck in a corner.

    :   **Parameters:**: `minChordLength` - the minimum length a chord must have to be considered valid. This should be a small positive number, like 1E-9 for example.

---


### setIterations

```
public void setIterations(int iterations)
```

:   Sets the total number of random points to be added to the provided
    results manager.

    :   **Parameters:**: `iterations` - the total number of random points to be added to the provided results manager. **Throws:**: `java.lang.IllegalArgumentException` - if `maxObstructedMoves` < 0

---


### setPointsPerPoint

```
public void setPointsPerPoint(int pointsPerPoint)
```

:   Sets the total number of points to generate for each point added to the
    provided results manager.

    :   **Parameters:**: `pointsPerPoint` - the total number of points to generate for each point added to the provided results manager. **Throws:**: `java.lang.IllegalArgumentException` - if `pointsPerPoint` < 1

---


### setMaxObstructedMoves

```
public void setMaxObstructedMoves(int maxObstructedMoves)
```

:   Sets the maximum number of obstructed moves to allow.

    :   **Parameters:**: `maxObstructedMoves` - the number of obstructed moves to allow. **Throws:**: `java.lang.IllegalArgumentException` - if `maxObstructedMoves` < 0

---


### getProgress

```
public ProgressMonitor getProgress()
```

:   Returns a progress monitor for this process.

    :   **Specified by:**: `getProgress` in interface `MonitoredProcess<CdHarComputer.Results>`
    :   **Returns:**: a progress monitor for this process.

---


### run

```
public void run(ResultsManager<CdHarComputer.Results> resultsManager)
```

:   Adds random points to the provided results manager.

    :   **Specified by:**: `run` in interface `KernelProcess<CdHarComputer.Results>`
    :   **Parameters:**: `resultsManager` - the results manager to which the random points are added. **Throws:**: `java.lang.IllegalStateException` - if any required information was not provided.


---


|  |  |  |
| --- | --- | --- |
| **Overview** \| **Package** \| **Class** \| **Use** \| **Tree** \| **Deprecated** \| **Index** \| **Help** | |  |
| PREV CLASS   NEXT CLASS | **FRAMES**    **NO FRAMES**     **All Classes** |  |
| SUMMARY: NESTED \| FIELD \| CONSTR \| METHOD | DETAIL: FIELD \| CONSTR \| METHOD |  |


---
